# Modelling seizure rates rather than time to an event within clinical trials of antiepileptic drugs

**DOI:** 10.1186/s12874-020-00965-5

**Published:** 2020-04-15

**Authors:** Laura J. Bonnett, Jane L. Hutton, Anthony G. Marson

**Affiliations:** 1 Department of Biostatistics, University of Liverpool, Waterhouse Building, Block F, 1-5 Brownlow Street, Liverpool, L69 3GL UK; 2 Department of Statistics, University of Warwick, Coventry, CV4 7AL UK; 3 Department of Molecular and Clinical Pharmacology, Clinical Sciences Centre, Aintree University Hospital, University of Liverpool, L9 7LJ & The Walton Centre NHS Foundation Trust, members of the Liverpool Health Partners, Lower Lane, Liverpool, UK

**Keywords:** Cox model, PWP-TT, Negative binomial, Epilepsy, Seizures

## Abstract

**Background:**

Predictive models within epilepsy are frequently developed via Cox’s proportional hazards models. These models estimate risk of a specified event such as 12-month remission. They are relatively simple to produce, have familiar output, and are useful to answer questions about short-term prognosis. However, the Cox model only considers time to first event rather than all seizures after starting treatment for example. This makes assessing change in seizure rates over time difficult. Variants to the Cox model exist enabling recurrent events to be modelled. One such variant is the Prentice, Williams and Peterson – Total Time (PWP-TT) model. An alternative is the negative binomial model for event counts. This study aims to demonstrate the differences between the three approaches, and to consider the benefits of the PWP-TT approach for assessing change in seizure rates over time.

**Methods:**

Time to 12-month remission and time to first seizure after randomisation were modelled using the Cox model. Risk of seizure recurrence was modelled using the PWP-TT model, including all seizures across the whole follow-up period. Seizure counts were modelled using negative binomial regression. Differences between the approaches were demonstrated using participants recruited to the UK-based multi-centre Standard versus New Antiepileptic Drug (SANAD) study.

**Results:**

Results from the PWP-TT model were similar to those from the conventional Cox and negative binomial models. In general, the direction of effect was consistent although the variables included in the models and the significance of the predictors varied. The confidence intervals obtained via the PWP-TT model tended to be narrower due to the increase in statistical power of the model.

**Conclusions:**

The Cox model is useful for determining the initial response to treatment and potentially informing when the next intervention may be required. The negative binomial model is useful for modelling event counts. The PWP-TT model extends the Cox model to all included events. This is useful in determining the longer-term effects of treatment policy. Such a model should be considered when designing future clinical trials in medical conditions typified by recurrent events to improve efficiency and statistical power as well as providing evidence regarding changes in event rates over time.

## Background

Epilepsy is defined as the tendency to have recurrent unprovoked seizures, and is one of the most prevalent chronic neurological conditions affecting approximately 70 million people worldwide [1]. In clinical practice a key aim of treatment is to achieve freedom from seizures with minimal adverse effects from antiepileptic drugs.

Standard internationally recognised outcomes in epilepsy include time to 12-month remission and time to treatment failure [2], and are most frequently modelled via Cox proportional hazards models. These models estimate risk of a specified event, are relatively simple to fit and have easily interpretable output. They are particularly useful to assess clinically relevant outcomes such as time to first seizure after commencement of treatment, as that is the time at which a change in treatment may happen.

The Cox model has notable disadvantages. In particular, it models time to one particular event after time zero such as time to 12-month remission from seizures, rather than modelling each seizure that occurs after randomisation in a clinical trial. Indeed, estimates suggest that 60 to 70% of people with epilepsy will achieve a remission from seizures [3]. However, up to 37% of people who achieve remission may proceed to have at least one further seizure whilst on antiepileptic drugs [4]. Considering time to first event only could limit the assessment of treatment policy and the ability to provide patients with an up-to-date prognosis following seizure occurrence.

When the event of interest, such as a seizure in epilepsy, can occur more than once in a participant, the events are termed recurrent events. Several approaches have been proposed to account for intra-subject correlation that arises from multiple events in survival analysis. These include variants to the Cox model [5, 6]. The most appropriate of these, based on the model assumptions and the clinical reality that seizures cluster and thus may not occur independently [7], is the Prentice, Williams and Peterson – Total Time (PWP-TT) model [8]. The PWP-TT model considers cumulative time since randomisation per event. An alternative is modelling event counts which can be done using negative binomial regression modelling.

Results from these three models have different interpretations. Cox models describe the risk of a specified event i.e. the first seizure after randomisation, or the first period of 12-month remission from seizures following randomisation. From a clinical perspective this is helpful to estimate when the next event of interest might happen from time zero. The PWP-TT and negative binomial models describe the rate of the event (i.e. number of events over a fixed time period) and can be used to assess the impact of longer-term policy on seizure frequency, as well as remission, within epilepsy.

The aim of this paper is to demonstrate the differences between the three approaches, and to highlight the benefits of the PWP-TT approach for assessing change in seizure rates over time. Included participants were those recruited to the UK-based multi-centre Standard versus New Antiepileptic Drug (SANAD) study.

## Methods

### Patients and procedures

Full details of the SANAD study are available in the original trial publications [9, 10]. Briefly, participants were eligible for randomisation into the SANAD study if they had a history of two or more clinically definite unprovoked epileptic seizures in the previous year. They were recruited to arm A if the recruiting clinician regarded carbamazepine the better standard treatment option than valproate, and arm B if the recruiting clinician regarded valproate the better standard treatment option than carbamazepine. In arm A, between 1st December 1999 and 1st June 2001, participants were allocated in a ratio of 1:1:1:1 to receive carbamazepine, gabapentin, lamotrigine or topiramate. From 1st June 2001 to 31st August 2004, an oxcarbazepine group was added to the trial and participants were randomly allocated in a ratio of 1:1:1:1:1 to receive carbamazepine, gabapentin, lamotrigine, oxcarbazepine, or topiramate. Within arm B, participants were allocated randomly in a 1:1:1 ratio to valproate, lamotrigine or topiramate between 12th January 1999 and 31st August 2004.

The primary outcomes across the SANAD study were time to treatment failure and time to 12 months of remission from seizures. Secondary outcomes included time to first post-randomisation seizure.

### Statistical Modelling

Cox’s proportional hazards regression model was used to model time to first seizure post randomisation and time to 12-month remission as these are frequently reported outcomes within the clinical literature. Full details of the methodology used to develop the prognostic model for time to 12-month remission for participants in SANAD have been reported previously [11, 12]. Identical methods were used for time to first seizure from randomisation. In brief, a pool of potential prognostic factors was established and a multivariable Cox model was derived by backwards selection according to Akaike’s Information Criterion [13]. Continuous variables were investigated using fractional polynomial transformations [14–17], and presented as post-hoc defined categorical variables with categories chosen based on knot positions for a spline model fit to the variable [18]. Similar methodology was used to model event counts, via the negative binomial model.

The PWP-TT model was used to estimate the rate of recurrent seizures based on data collected over the full study. It is a multiple time-to-event approach to modelling the data and accounts for missing data via censoring [19]. It assumes that subjects cannot be at risk for say a fourth seizure until they have a third seizure, which is a valid clinical assumption within epilepsy. The PWP-TT model enables inclusion of all post-randomisation seizures, not just the first for example. As for the Cox models, variables from the pool of potential prognostic factors were included in a multivariable model via backwards selection according to Akaike’s Information Criterion, [13] and continuous covariates were assessed for best fit.

The list of possible prognostic factors for inclusion in the models was developed based on clinical consensus and previous related publications [20, 21]: gender, febrile seizure history, first degree relative with epilepsy, treatment history (antiepileptic drug treatment prior to randomisation), age at randomisation, annual rate of tonic-clonic seizures prior to randomisation (total number of tonic-clonic seizures prior to randomisation divided by time from first seizure to randomisation), neurological insult (learning disabilities or a neurological deficit), electroencephalogram (EEG) result, and seizure type. EEG result was classified as normal, not done, non-specific abnormality, or epileptiform abnormality (focal or generalised spikes, or spike and slow wave activity). Additionally, focal epilepsy site of onset and CT or MRI scan result were also included in the pool of possible factors for arm A of the study.

Treatment was forced into each model as all participants were treated at randomisation. As only 44 participants were classified as having generalised epilepsy in arm A, and only 54 participants were classified as having focal epilepsy in arm B these participants were excluded from this analysis. Therefore arm A included 1491 participants with focal epilepsy and 157 with unclassified epilepsy, and arm B included 464 participants with generalised epilepsy and 184 participants with unclassified epilepsy. The two arms were modelled separately.

In the development of prognostic models for time to 12-month remission and time to treatment failure previously [11, 22], stratification was used to account for the late addition of oxcarbazepine to the arm A of the study. However, this was found to have little effect on the results [11] and so the stratification term was dropped to ensure that all drugs could be included in the PWP-TT and negative binomial models.

The initial comparison between models used the data from arm A as it was the largest dataset. However, arm B data was also considered to determine the generalisability of the results. A number of sensitivity analyses were also considered, again to determine the generalisability of the results. In particular, in SANAD clinicians were free to prescribe any treatment they deemed appropriate after withdrawal of the randomised drug. Therefore, the dataset includes many possible drug combinations which adds complexity to the statistical model. Therefore two approaches were taken; include everyone, and censor people at the time when they come off their randomised drug. Additionally, sensitivity analyses considered recurrent tonic-clonic seizures only (arm A and B) and recurrent tonic-clonic and complex partial seizures only (arm A).

## Results

### Seizure characteristics

**Fig. 1:**
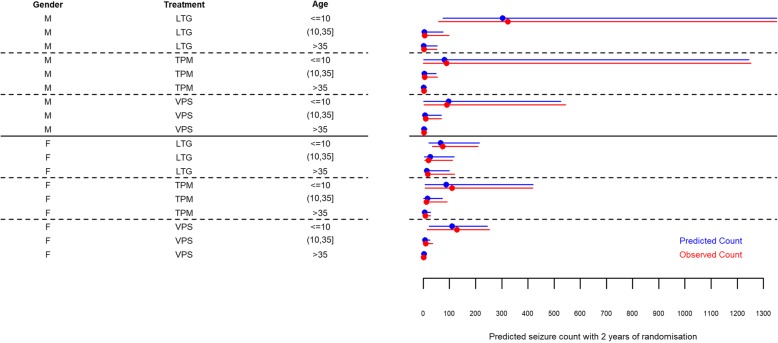
Box and whisker plots for total non-zero within-study seizures by seizure type, by study arm. SP: simple partial; CP: complex partial; SCGTC: simple or complex partial with generalised tonic-clonic; M: myoclonic; TA: typical absence; AA: atypical absence; TC: generalised tonic-clonic; OTC: other tonic-clonic

Outcome data were available for 1648 participants in arm A and 637 in arm B. 443 arm A participants had zero seizures during follow-up and of these, 380 (86%) people were classified as having focal epilepsy. 200 arm B participants had zero seizures during follow-up and of these, 123 (62%) people were classified as having generalised epilepsy. The annual rate of seizures, per seizure type, for participants with seizures post-randomisation in arms A and B can be seen in Fig. 1. Arm A participants had a median rate of about 10 seizures per year across the three seizure types. Arm B groups were more variable, but generally had higher seizure frequency.

### Arm A – focal epilepsy

**Fig. 2:**
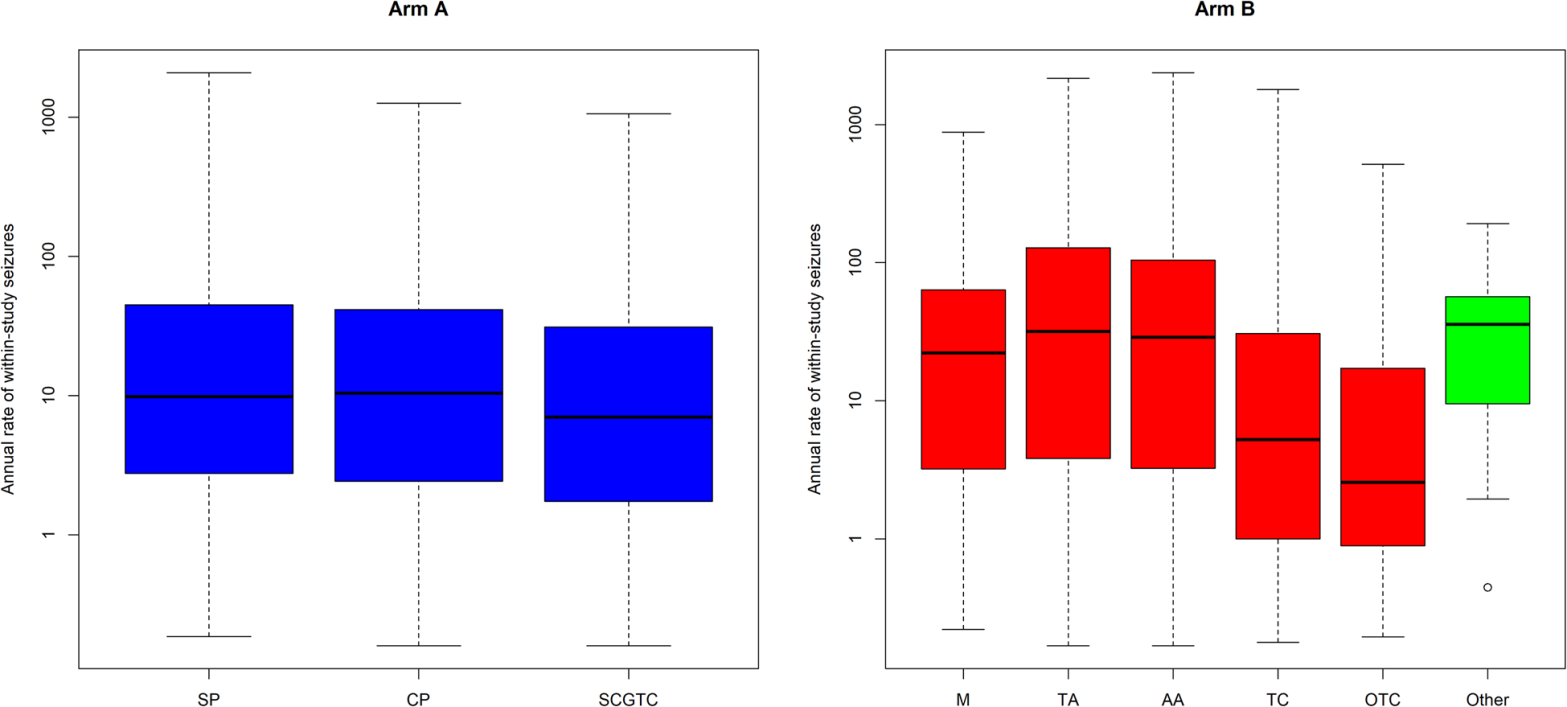
Visualisation of the Cox and PWP-TT models according to treatment group – arm A. Black lines and crosses shows median time to first seizure estimated from a Cox model, red lines and crosses represent randomly generated event times according the predicted number of events from the PWP-TT model

Table 1 summarises the effect of treatment on outcome according to each of the four models - two Cox, negative binomial and the PWP-TT. The difference in interpretation between the Cox and PWP-TT models can be illustrated graphically as in Fig. 2. This figure was generated based on the median time to first seizure per treatment group in the case of the Cox model (shown in black), and randomly generated times from the uniform distribution based on the median number of predicted seizures per treatment group based on the PWP-TT model (shown in red). Although carbamazepine, oxcarbazepine and topiramate have the longest times to first seizure, in the longer term oxcarbazepine shows a lower number of seizures than both carbamazepine and topiramate. As a rate of zero indicates remission, lower average seizures rates imply more people achieving remission.

**Table 1:** Risk of seizure recurrence by treatment – arm A

Variable		Hazard Ratio (95% CI)			Negative binomial: rate of seizures
		PWP-TT: Rate of recurrent seizures	Cox: First seizure	Cox: 12-month remission	
Treatment	Carbamazepine	1.00	1.00	1.00	1.00
	Gabapentin	0.97 (0.89, 1.07)	1.34 (1.13, 1.59)	0.76 (0.63, 0.91)	1.45 (1.06, 1.99)
	Lamotrigine	0.98 (0.89, 1.08)	1.23 (1.04, 1.45)	0.91 (0.76, 1.09)	0.91 (0.67, 1.25)
	Oxcarbazepine	0.91 (0.82, 1.02)	1.03 (0.84, 1.26)	1.03 (0.83, 1.28)	0.67 (0.46, 0.98)
	Topiramate	1.06 (0.97, 1.17)	1.06 (0.90, 1.26)	0.84 (0.70, 1.01)	1.25 (0.91, 1.72)
Intercept		N/A	N/A	N/A	−2.68 (−2.89, − 2.45)

According to the Cox models (Table 1), risk of first seizure and chance of not achieving 12-month remission are significantly higher on gabapentin than carbamazepine – chance of not achieving 12-month remission is equivalent to 1 divided by the chance of the event. A short time to first seizure implies a higher chance of not achieving 12 month remission, which is reflected in the two Cox models estimating gabapentin and lamotrigine to be less effective than carbamazepine. Median time to first seizure on lamotrigine is 37 days shorter than carbamazepine and median time to remission is 120 days shorter on carbamazepine than on lamotrigine. These results are broadly in line with the negative binomial model which shows rate of seizures is significantly higher on gabapentin than carbamazepine and significantly lower on oxcarbazepine than carbamazepine.

According to the PWP-TT model (Table 1), people on topiramate have a 6% higher rate of recurrent seizures than those on carbamazepine. The direction of the effect is generally opposite to that for the Cox models and none of the results are significant. Therefore, taking all four models into consideration, gabapentin and lamotrigine appear worse at delaying a first seizure than carbamazepine. In the longer term there is less difference between the treatment policies because if the first treatment does not work, it will be changed, and if necessary changed again, aiming for seizure control. The PWP-TT model better captures the longer term consequence of this treatment policy. The size of effect is closer to zero with narrower confidence intervals for the PWP-TT model than the results seen with the Cox and negative binomial models. This is because the statistical power is maximised in the PWP-TT model [19].

The multivariable PWP-TT model included all potential covariates except for age; the Cox and negative binomial models included fewer covariates as shown in Table 2. As the multivariable PWP-TT model is more powerful for rate of recurrent seizures, more potential covariates with narrower confidence intervals are included than with either Cox model. The Cox models did not include febrile seizure history, first degree relative with epilepsy and EEG result. The negative binomial model did include EEG result but additionally neurological insult, focal site of onset, and annual rate of seizures prior to randomisation were excluded from the model. No drug has significantly higher seizure rates than carbamazepine, but gabapentin was significant in both Cox models and gabapentin was significant in the negative binomial model.

**Table 2:** PWP-TT, Cox and negative binomial models for participants in arm A of SANAD

Variable		Hazard Ratio (95% CI)			Negative Binomial: Rate of seizures
		PWP-CP: Rate of recurrent seizures	Cox PH: First seizure	Cox PH: 12-month remission	
Gender	Female Male	1.00 0.94 (0.89, 1.00)	1.00 0.84 (0.75,0.94)	1.00 1.22 (1.07, 1.38)	1.00 0.72 (0.59, 0.89)
Febrile Seizure History	Absent Present	1.00 1.06 (0.93, 1.20)	N/A	N/A	N/A
First degree relative with epilepsy	Absent Present	1.00 0.88 (0.80, 0.97)	N/A	N/A	N/A
Treatment History	Treatment naïve Seizures after remission Taking non-SANAD AEDs	1.00 0.92 (0.70, 1.22) 1.04 (0.96, 1.11)	1.00 1.02 (0.70, 1.47) 1.59 (1.37, 1.85)	1.00 0.87 (0.58, 1.30) 0.52 (0.43, 0.63)	1.00 0.99 (0.52,2.15) 1.86 (1.14, 2.51)
Neurological Insult	Absent Present	1.00 1.07 (0.98, 1.17)	1.00 1.20 (1.01, 1.42)	1.00 0.78 (0.63, 0.97)	N/A
EEG Result	Normal Epileptiform abnormality Non-specific abnormality Not clinically indicated	1.00 1.01 (0.94, 1.09) 1.01 (0.93, 1.11) 0.95 (0.85, 1.07)	N/A	N/A	1.00 1.24 (0.86, 1.82) 0.69 (0.51, 0.94) 0.95 (0.73, 1.23)
CT or MRI Result	Normal Abnormal Not clinically indicated	1.00 1.10 (1.02, 1.18) 0.93 (0.85, 1.02)	N/A	1.00 0.89 (0.77, 1.04) 1.16 (0.97, 1.38)	N/A
Focal site of onset	Temporal Not localised Frontal Other Unclassified	1.00 0.98 (0.91, 1.05) 0.84 (0.73, 0.96) 0.83 (0.71, 0.96) 0.50 (0.29, 0.86)	N/A	1.00 0.93 (0.80, 1.07) 1.18 (0.91, 1.54) 1.26 (0.97, 1.65) 1.33 (1.07, 1.65)	N/A
Age at randomisation (years)	≤10 11–24 25–36 37–49 50–70 ≥71	N/A	1.00 0.99 (0.99, 1.00) 0.99 (0.98, 0.99) 0.98 (0.97, 0.99) 0.97 (0.96, 0.99) 0.97 (0.95, 0.99)	1.00 1.01 (1.00, 1.01) 1.01 (1.00, 1.02) 1.02 (1.01, 1.03) 1.03 (1.01, 1.04) 1.03 (1.01, 1.05)	1.00 0.91 (0.86, 0.96) 0.81 (0.71, 0.92) 0.71 (059, 0.87) 0.61 (0.45, 0.82) 0.51 (0.34, 0.76)
Annual rate seizures prior to randomisation	≤1 1–4 4–10 10–25 25–175 ≥175	1.00 0.98 (0.96, 0.99) 0.96 (0.94, 0.99) 0.95 (0.92, 0.98) 0.93 (0.88, 0.97) 0.89 (0.83, 0.96)	1.00 1.15 (1.12, 1.18) 1.26 (1.21, 1.32) 1.37 (1.30, 1.45) 1.61 (1.48, 1.75) 2.06 (1.82, 2.34)	1.00 0.86 (0.83, 0.90) 0.75 (0.69, 0.81) 0.60 (0.52, 0.69) 0.52 (0.43, 0.63) 0.45 (0.36, 0.56)	N/A
Seizure type	Simple or complex partial Simple or complex partial with generalised tonic-clonic Uncertain	1.00 1.09 (1.02, 1.17) 1.91 (1.15, 3.17)	1.00 0.93 (0.81, 1.05) 0.67 (0.54, 0.84)	N/A	1.00 0.75 (0.59, 0.95) 1.19 (0.26, 15.42)
Treatment	Carbamazepine Gabapentin Lamotrigine Oxcarbazepine Topiramate	1.00 0.97 (0.88, 1.06) 0.98 (0.89, 1.08) 0.92 (0.82, 1.02) 1.06 (0.97, 1.17)	1.00 1.42 (1.20, 1.68) 1.26 (1.07, 1.50) 1.11 (0.90, 1.36) 1.08 (0.91, 1.28)	1.00 0.73 (0.60, 0.87) 0.89 (0.74, 1.06) 0.98 (0.79, 1.22) 0.82 (0.68, 0.99)	1.00 1.41 (1.03, 1.93) 1.02 (0.74, 1.39) 0.80 (0.56, 1.17) 1.13 (0.83, 1.53)
Intercept		N/A	N/A	N/A	−2.17 (−2.62, −1.71)

N/A – variable not included in multivariable model

The PWP-TT model estimated a 10% lower seizure recurrence rate among people with simple or complex partial seizures only compared to those with simple or complex partial seizures and generalised tonic-clonic seizures. People with uncertain seizure types had long-term seizure rates almost twice that of people with simple or complex partial seizures only. The results also suggest that people with a low rate of seizures prior to randomisation have a higher rate of recurrent seizures than those with higher rates prior to randomisation.

The direction of the effect for most statistically significant variables was the same across models, with the PWP-TT coefficients shrunk towards one. Differences in direction of estimated effect are likely due to the different variables include in the multivariable model and the resulting effect on the interactions between these variables.

**Fig. 3:**
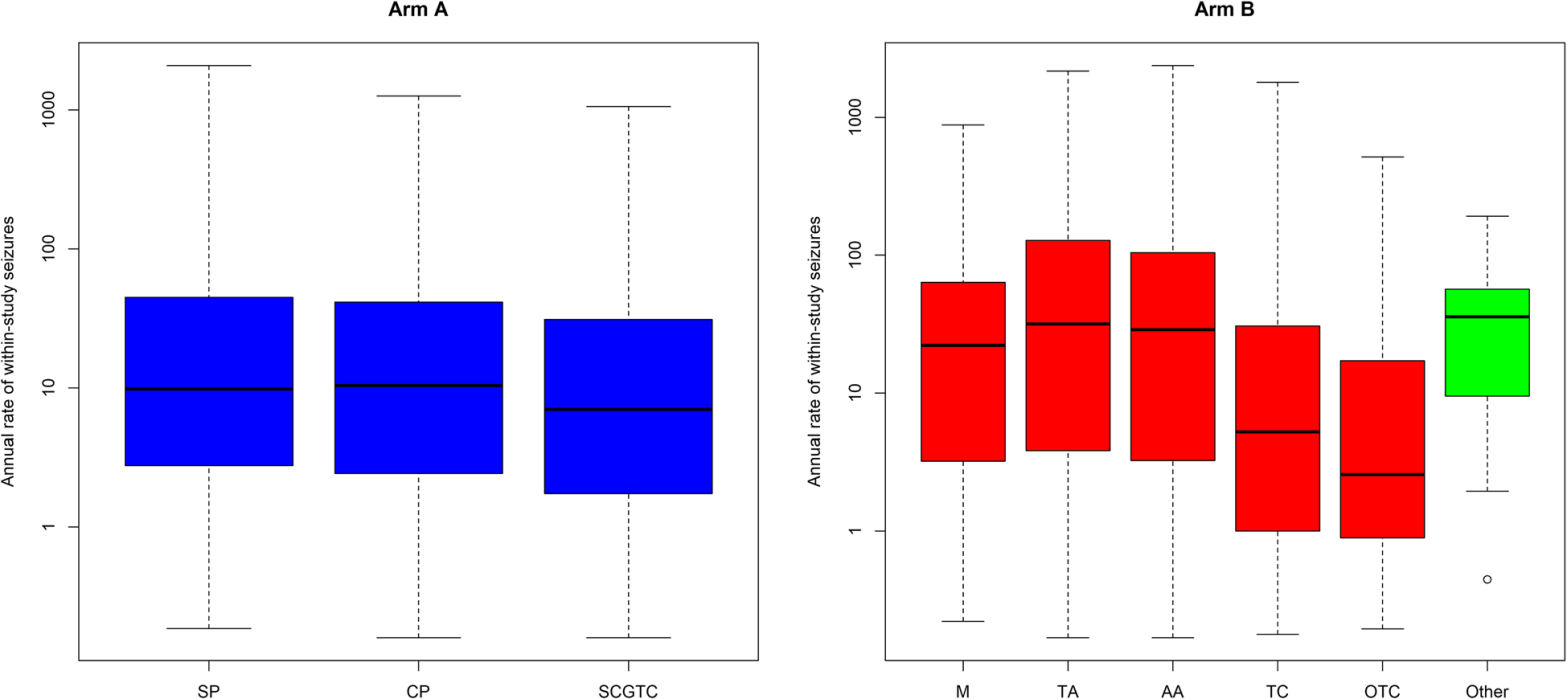
Seizure counts from the PWP-TT model based on combinations of risk factors (arm A). Circles show median seizure counts while lines show interquartile ranges of seizure counts

A forest plot comparing median seizure counts (in blue) predicted from the PWP-TT model according to combinations of patient characteristics can be seen in Fig. 3. The associated observed seizure counts are also included for comparison (in red). Gender is the most influential factor with women having higher predicted and observed seizure counts within 2 years of randomisation than men, and thus lowest chance of remission.

### Arm B – generalised epilepsy

For comparison, identical analyses were considered using the data from arm B. Table 3 summarises the effect of treatment on outcome according to each of the four models.

**Table 3:** Risk of seizure recurrence by treatment – arm B

Variable		Hazard Ratio (95% CI)			Negative binomial: Rate of seizures
		PWP-TT: Rate of recurrent seizures	Cox: First seizure	Cox: 12-month remission	
Treatment	Valproate	1.00	1.00	1.00	1.00
	Lamotrigine	1.12 (0.99, 1.26)	1.40 (1.12, 1.76)	0.75 (0.60, 0.93)	1.86 (1.15, 2.99)
	Topiramate	1.13 (1.00, 1.29)	1.21 (0.97, 1.52)	0.87 (0.70, 1.08)	1.66 (1.03, 2.67)
Intercept		N/A	N/A	N/A	−2.42 (− 2.74, − 2.07)

**Fig. 4:**
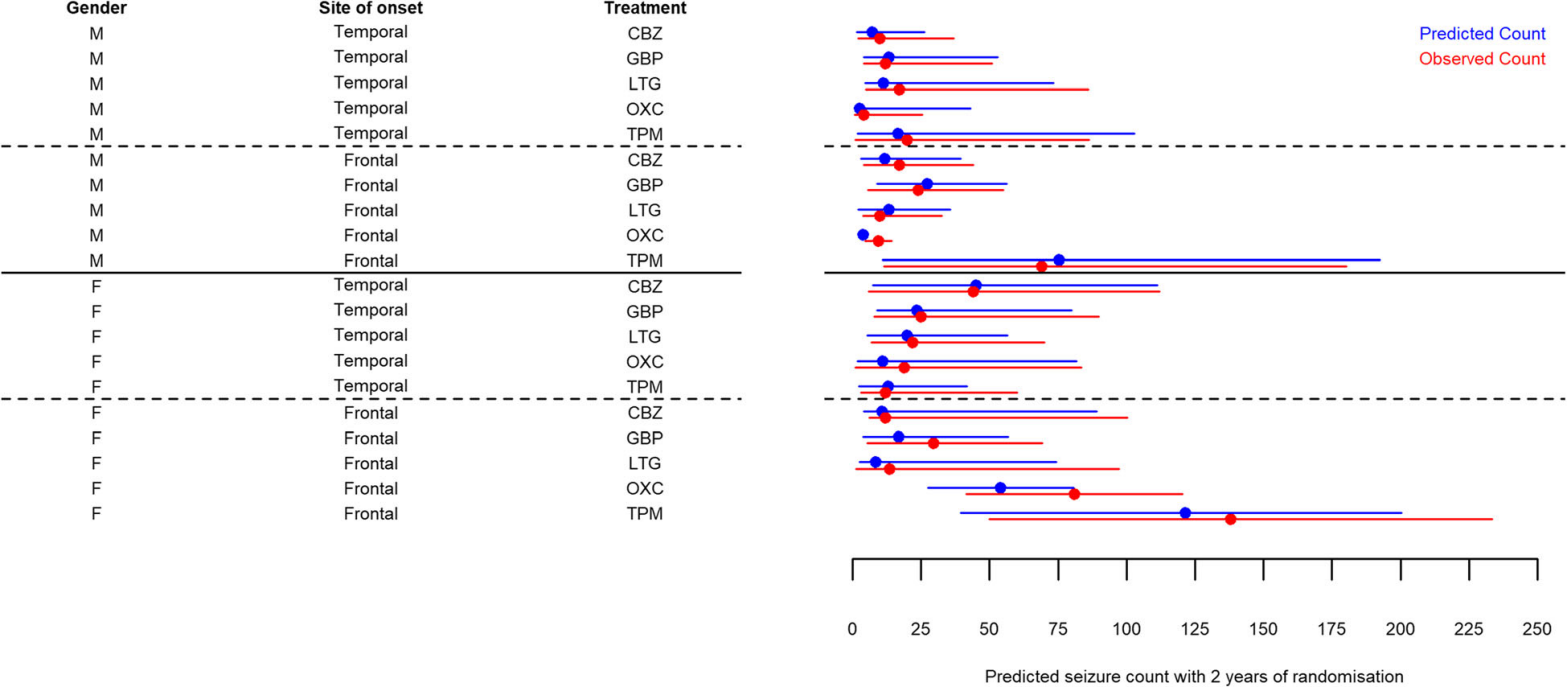
Visualisation of the Cox and PWP-TT models according to treatment group – arm B. Black lines and crosses show median time to first seizure according to a Cox model; red lines and crosses represent randomly generated event times according the predicted number of events from the PWP-TT model

The difference in interpretation between the Cox and PWP-TT models is again illustrated graphically, in Fig. 4. Although valproate has a slightly longer median time to remission than topiramate, in the longer term both valproate and topiramate lead to a lower number of seizures than lamotrigine. According to the Cox models, risk of first seizure and chance of not achieving 12-month remission (1/chance of remission) are significantly higher on lamotrigine than valproate. A short time to first seizure implies a higher chance of not achieving 12 month remission, which is reflected in the two Cox models estimating lamotrigine to be less effective than valproate. Rate of seizures are also higher on lamotrigine and topiramate than valproate according to the negative binomial model.

According to the PWP-TT model, people on lamotrigine have a 12% higher rate of recurrent seizures than those on valproate on average, and 13% higher in participants on topiramate than on valproate. The direction of the effect is in agreement with that from the Cox models but only the result for topiramate is significant. Therefore, taking all four models into consideration, valproate is much better at delaying a first seizure than lamotrigine and topiramate, but in the longer term there is less difference in the effect of treatment policies on seizure rate and remission. Again, the size of the effect is closer to zero with narrower confidence intervals for the PWP-TT than the results seen with the Cox and negative binomial models as the power is maximised.

As the multivariable PWP-TT model is more powerful for rate of recurrent seizures, more potential covariates with narrower confidence intervals are included than with either Cox model (Table 4). The Cox models did not include febrile seizure history or EEG result. The negative binomial included febrile seizure history but additionally excluded gender and annual rate of seizures prior to randomisation. Topiramate had significantly higher seizure rates than valproate, but was not significant in the Cox or negative binomial models.

**Table 4:** PWP-TT, Cox and negative binomial models for participants in arm B of SANAD

Variable		Hazard Ratio (95% CI)			Negative binomial: Rate of seizures
		PWP-CP: Rate of recurrent seizures	Cox PH: First seizure	Cox PH: 12-month remission	
Gender	Female Male	1.00 1.09 (0.99, 1.20)	1.00 0.79 (0.65,0.96)	N/A	N/A
Febrile Seizure History	Absent Present	1.00 0.72 (0.63, 0.82)	N/A	N/A	1.00 0.43 (0.22, 0.91)
First degree relative with epilepsy	Absent Present	1.00 1.11 (0.96, 1.28)	1.00 1.28 (1.01, 1.61)	1.00 0.71 (0.56, 0.91)	1.00 2.04 (1.30, 3.31)
Treatment History	Treatment naïve Seizures after remission Taking non-SANAD AEDs	1.00 0.74 (0.59, 0.92) 1.18 (0.99, 1.41)	N/A	1.00 0.85 (0.52, 1.41) 0.79 (0.49, 1.00)	1.00 0.77 (0.30, 2.36) 3.40 (1.75, 7.30)
Neurological Insult	Absent Present	1.00 1.38 (1.23, 1.55)	1.00 1.27 (0.95, 1.70)	1.00 0.64 (0.47, 0.87)	1.00 2.84 (1.56, 5.53)
EEG Result	Normal Epileptiform abnormality Non-specific abnormality Not clinically indicated	1.00 1.02 (0.90, 1.15) 0.83 (0.67, 1.03) 0.78 (0.61, 0.99)	N/A	N/A	N/A
Treatment	Valproate Lamotrigine Topiramate	1.00 1.17 (1.05, 1.31) 1.16 (1.02, 1.31)	1.00 1.53 (1.22, 1.93) 1.23 (0.98, 1.55)	1.00 0.79 (0.64, 0.99) 0.92 (0.74, 1.14)	1.00 1.02 (0.65, 1.61) 1.51 (0.96, 2.38)
Age at randomisation (years)	≤7 8–13 14–19 20–27 28–50 > 50	1.00 0.97 (0.95, 0.99) 0.95 (0.90, 0.99) 0.93 (0.87, 0.99) 0.90 (0.82, 0.98) 0.87 (0.78, 0.97)	1.00 0.93 (0.89, 0.97) 0.87 (0.80, 0.95) 0.83 (0.74, 0.93) 0.77 (0.66, 0.91) 0.72 (0.59, 0.89)	N/A	1.00 0.82 (0.77, 0.88) 0.62 (0.53, 0.72) 0.44 (0.34, 0.57) 0.21 (0.12, 0.35) 0.07 (0.02, 0.16)
Annual rate of tonic-clonic seizures prior to randomisation	≤1 1–2 2–6 > 6	N/A	1.00 1.02 (0.99, 1.06) 1.05 (0.98, 1.11) 1.09 (0.97, 1.21)	N/A	N/A
Seizure type	Generalised tonic-clonic Absence Myoclonic or absence with tonic-clonic Unclassified tonic-clonic Other seizures	1.00 0.85 (0.72, 1.01) 1.05 (0.90, 1.23) 1.05 (0.90, 1.23) 0.87 (0.74, 1.03)	1.00 3.34 (1.98, 5.63) 2.18 (1.67, 2.84) 1.16 (0.88, 1.53) 2.63 (1.70, 4.08)	1.00 0.62 (0.47, 0.81) 0.56 (0.43, 0.72) 0.83 (0.65, 1.05) 0.58 (0.39, 0.86)	1.00 1.52 (6.70, 21.16) 6.34 (3.82, 10.61) 1.42 (0.88, 2.32) 4.77 (2.22, 11.42)
Intercept		N/A	N/A	N/A	−3.03 (−3.77, −2.77)

N/A – variable not included in multivariable model

The The PWP-TT model estimated estimated a 28% lower rate of recurrent seizures among people with a history of febrile seizures than those who did not have such a history, and participants with seizures after a period of remission had a lower rate than treatment naïve participants. People with a neurological insult and those on lamotrigine or topiramate had higher rates of recurrent seizures than those on valproate. Older participants (over 50) had seizure recurrence rates 13% lower than those aged less than eight.

The PWP-TT results imply that valproate has a longer expected time to first seizure and shorter time to remission. The direction of the effect for most statistically significant variables was the same across models with the PWP-TT coefficients shrunk towards one. Differences in direction of estimated effect are again likely due to the different variables included in the multivariable model and the resulting effect on the interactions between these variables.

**Fig. 5:**
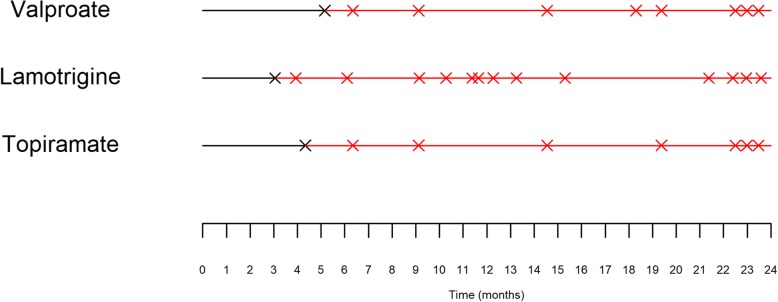
Median and quartiles for seizure counts from the PWP-TT model for Arm B based on combinations of risk factors. All participants are assumed to have no history of febrile seizures

A forest plot comparing median seizure counts (in blue) predicted from the PWP-TT model according to combinations of patient characteristics can be seen in Fig. 5. The associated observed seizure counts are also included for comparison (in red). Age is the most influential factor with the youngest people having the highest predicted seizure count within 2 years of randomisation, and thus lowest chance of remission.

### Sensitivity analyses – PWP-TT model

#### Arm A – focal epilepsy

Sensitivity analyses of the PWP-TT model with censoring at withdrawal of randomised drug, and based on specific recurrent seizure types for patients in arm A can be seen in Table 5. The results for recurrent tonic-clonic seizures only, and recurrent tonic-clonic and complex partial seizures only are the same suggesting that the predicted rate of these seizures is similar. Censoring at withdrawal of randomised drug has little effect on the results, although the direction of effect for gabapentin changes and the results for gabapentin and topiramate become significant when the model is unadjusted for any other variables.

**Table 5:** Risk of seizure recurrence by treatment - sensitivity analyses (arm A)

		Hazard Ratio (95% CI)				
Variable		**On randomised drug only**	**Recurrent tonic-clonic recurrent seizures only**	**On randomised drug only & recurrent tonic-clonic seizures only**	**Recurrent tonic-clonic & complex partial seizures only**	**On randomised drug only & recurrent tonic-clonic & complex partial seizures only**
Treatment	Carbamazepine	1.00	1.00	1.00	1.00	1.00
	Gabapentin	1.19 (1.05, 1.36)	1.01 (0.87, 1.17)	1.02 (0.85, 1.23)	1.01 (0.87, 1.17)	1.02 (0.85, 1.23)
	Lamotrigine	0.94 (0.83, 1.07)	0.97 (0.84, 1.13)	0.83 (0.69, 0.99)	0.97 (0.84, 1.13)	0.83 (0.69, 0.99)
	Oxcarbazepine	0.83 (0.71, 0.97)	0.87 (0.71, 1.05)	0.75 (0.59, 0.96)	0.87 (0.71, 1.05)	0.75 (0.59, 0.96)
	Topiramate	1.18 (1.03, 1.34)	1.02 (0.87, 1.19)	0.99 (0.83, 1.19)	1.02 (0.87, 1.19)	0.99 (0.83, 1.19)

The results for the multivariable PWP-TT models according to each sensitivity analysis can be seen in Table 6. In general fewer variables were included in the multivariable models than seen in the original PWP-TT model. The direction of effect is generally consistent with the original results.

**Table 6:** Sensitivity analysis of the PWP-TT model (arm A)

Variable		Hazard Ratio (95% CI)				
		On randomised drug only	Recurrent tonic-clonic seizures only	On randomised drug only & recurrent tonic-clonic seizures only	Recurrent tonic-clonic & complex partial seizures only	On randomised drug only & recurrent tonic-clonic & complex partial seizures only
Gender	Female Male	N/A	1.00 0.88 (0.85, 0.91)	1.00 0.84 (0.80, 0.88)	1.00 0.88 (0.85, 0.91)	1.00 0.84 (0.80, 0.88)
Febrile Seizure History	Absent Present	N/A	N/A	1.00 1.11 (0.99, 1.25)	N/A	1.00 1.11 (0.99, 1.25)
First degree relative with epilepsy	Absent Present	N/A	1.00 0.88 (0.84, 0.93)	1.00 0.93 (0.86, 1.00)	1.00 0.88 (0.84, 0.93)	1.00 0.93 (0.86, 1.00)
Treatment History	Treat. naïve Seizures Not SANAD AED	1.00 0.94 (0.85, 1.03) 1.05 (1.01, 1.09)	1.00 0.77 (0.70, 0.86) 1.01 (0.98, 1.05)	1.00 1.30 (1.11, 1.52) 1.02 (0.96, 1.08)	1.00 0.77 (0.70, 0.86) 1.01 (0.98, 1.05)	1.00 1.30 (1.11, 1.52) 1.02 (0.96, 1.08)
Neurological Insult	Absent Present	N/A	N/A	N/A	N/A	N/A
EEG Result	Normal Epi. abnorm. N/S abnorm. Not indicated	1.00 0.80 (0.76, 0.84) 0.88 (0.84, 0.92) 1.04 (1.01, 1.08)	1.00 0.94 (0.88, 1.00) 0.93 (0.88, 0.98) 0.97 (0.93, 1.02)	1.00 0.93 (0.83, 1.03) 0.90 (0.84, 0.97) 1.09 (1.02, 1.16)	1.00 0.94 (0.88, 1.00) 0.93 (0.88, 0.98) 0.97 (0.93, 1.02)	1.00 0.93 (0.83, 1.03) 0.90 (0.84, 0.97) 1.09 (1.02, 1.16)
CT or MRI Result	Normal Abnormal Not indicated	1.00 1.05 (1.01, 1.08) 0.95 (0.91, 0.99)	1.00 1.17 (1.12, 1.22) 1.10 (1.04, 1.16)	1.00 1.03 (0.98, 1.10) 1.16 (1.08, 1.25)	1.00 1.17 (1.12, 1.22) 1.10 (1.04, 1.16)	1.00 1.03 (0.98, 1.10) 1.16 (1.08, 1.25)
Focal site of onset	Temporal Not localised Frontal Other Unclassified	1.00 1.06 (1.03, 1.10) 1.29 (1.22, 1.37) 0.84 (0.79, 0.90) 0.43 (0.30, 0.60)	1.00 1.05 (1.01, 1.09) 0.93 (0.86, 1.00) 1.01 (0.93, 1.09) 0.39 (0.30, 0.50)	1.00 0.98 (0.93, 1.04) 0.85 (0.76, 0.96) 0.94 (0.84, 1.05) 0.44 (0.29, 0.67)	1.00 1.05 (1.01, 1.09) 0.93 (0.86, 1.00) 1.01 (0.30, 0.50) 0.39 (0.93, 1.02)	1.00 0.98 (0.93, 1.04) 0.85 (0.76, 0.96) 0.94 (0.84, 1.05) 0.44 (0.29, 0.67)
Age at randomisation (years)	≤10 11–24 25–36 37–49 50–70 ≥71	1.00 1.01 (1.00, 1.01) 1.01 (1.00, 1.02) 1.02 (1.00, 1.03) 1.02 (1.00, 1.05) 1.03 (1.00, 1.06)	N/A	N/A	N/A	N/A
Annual rate seizures prior to randomisation	1 1–4 4–10 10–25 25–175 ≥175	1.00 1.01 (1.00, 1.01) 1.01 (1.00, 1.02) 1.02 (1.00, 1.03) 1.03 (1.00, 1.05) 1.04 (1.01, 1.07)	N/A	1.00 1.04 (1.02, 1.05) 1.06 (1.04, 1.08) 1.08 (1.05, 1.11) 1.13 (1.08, 1.17) 1.17 (1.11, 1.23)	N/A	1.00 1.04 (1.02, 1.05) 1.06 (1.04, 1.08) 1.08 (1.05, 1.11) 1.13 (1.08, 1.17) 1.17 (1.11, 1.23)
Seizure type	S/C partial S/C + gen. TC Uncertain	1.00 0.96 (0.94, 0.99) 2.05 (1.47, 2.87)	1.00 0.84 (0.80, 0.88) 2.03 (1.57, 2.62)	1.00 1.05 (0.98, 1.13) 2.06 (1.36, 3.13)	1.00 0.84 (0.80, 0.88) 2.03 (1.57, 2.62)	1.00 1.05 (0.98, 1.13) 2.06 (1.36, 3.13)
Treatment	Carbamazepine Gabapentin Lamotrigine Oxcarbazepine Topiramate	1.00 1.20 (1.15, 1.25) 0.94 (0.90, 0.97) 0.84 (0.79, 0.89) 1.15 (1.11, 1.20)	1.00 0.98 (0.93, 1.03) 0.95 (0.90, 1.01) 0.85 (0.80, 0.91) 0.97 (0.80, 0.88)	1.00 1.05 (0.97, 1.14) 0.86 (0.80, 0.93) 0.75 (0.68, 0.83) 1.01 (0.93, 1.09)	1.00 0.98 (0.93, 1.03) 0.95 (0.90, 1.01) 0.85 (0.80, 0.91) 0.97 (0.92, 1.03)	1.00 1.05 (0.97, 1.14) 0.86 (0.80, 0.93) 0.75 (0.68, 0.83) 1.01 (0.93, 1.09)

N/A – variable not included in multivariable model; Epi. abnorm. – epileptiform abnormality;

N/S abnorm. – non-specific abnormality; S/C – simple or complex; gen. TC – generalised tonic-clonic

#### Arm B – generalised epilepsy

Sensitivity analyses of the PWP-TT model with censoring at withdrawal of randomised drug, and based on specific recurrent seizure types for patients in arm B can be seen in Table 7. The varying conditions had little effect on the results which maintain their significance and direction. The results for the multivariable PWP-TT models according to each sensitivity analysis can be seen in Table 8. In general fewer variables were included in the models than seen originally. The direction of effect is generally consistent with the original results.

**Table 7:** Risk of seizure recurrence by treatment - sensitivity analyses (arm B)

Variable		Hazard Ratio (95% CI)		
		On randomised drug only	Recurrent tonic-clonic seizures only	On randomised drug only & recurrent tonic-clonic seizures only
Treatment	Valproate	1.00	1.00	1.00
	Lamotrigine	1.18 (1.01, 1.38)	1.26 (1.06, 1.51)	1.54 (1.23, 1.93)
	Topiramate	1.58 (1.33, 1.88)	1.24 (1.03, 1.49)	2.08 (1.64, 2.63)

**Table 8:** Sensitivity analysis of the PWP-TT model (arm B)

Variable		Hazard Ratio (95% CI)		
		On randomised drug only	Recurrent tonic-clonic seizures only	On randomised drug only & recurrent tonic-clonic seizures only
Gender	Female Male	1.00 1.29 (1.22, 1.36)	1.00 1.29 (1.19, 1.39)	1.00 1.28 (1.14, 1.44)
Febrile Seizure History	Absent Present	N/A	1.00 0.82 (0.71, 0.95)	1.00 1.33 (1.09, 1.63)
First degree relative with epilepsy	Absent Present	1.00 1.21 (1.14, 1.30)	N/A	1.00 1.55 (1.37, 1.75)
Treatment History	Treat. naïve Seizures Not SANAD AED	1.00 0.81 (0.68, 0.97) 1.45 (1.31, 1.61)	1.00 0.68 (0.53, 0.87) 1.08 (0.97, 1.21)	1.00 1.09 (0.79, 1.50) 1.46 (1.24, 1.72)
Neurological Insult	Absent Present	N/A	1.00 1.17 (1.04, 1.31)	1.00 0.79 (0.66, 0.93)
EEG Result	Normal Epi. abnorm. N/S abnorm. Not indicated	1.00 0.77 (0.64, 0.93) 0.75 (0.65, 0.85) 1.19 (1.11, 1.29)	1.00 0.74 (0.63, 0.87) 0.61 (0.53, 0.70) 0.83 (0.76, 0.91)	1.00 1.01 (0.75, 1.35) 0.65 (0.54, 0.80) 1.08 (0.95, 1.23)
Age at randomisation (years)	≤7 8–13 14–19 20–27 28–50 > 50	1.00 0.94 (0.93, 0.95) 0.89 (0.87, 0.91) 0.85 (0.82, 0.88) 0.80 (0.76, 0.84) 0.75 (0.71, 0.80)	1.00 0.96 (0.94, 0.97) 0.92 (0.88, 0.95) 0.89 (0.84, 0.93) 0.85 (0.79, 0.91) 0.81 (0.74, 0.89)	1.00 0.98 (0.95, 1.00) 0.96 (0.91, 1.01) 0.94 (0.88, 1.01) 0.92 (0.84, 1.01) 0.90 (0.80, 1.02)
Annual rate seizures prior to randomisation	1 1–2 2–6 > 6	N/A	1.00 0.99 (0.98, 1.00) 0.98 (0.96, 1.00) 0.96 (0.92, 0.99)	N/A
Seizure type	Gen. TC Absence Myo./Abs + TC Unclass. TC Other	1.00 1.14 (1.04, 1.26) 1.12 (1.03, 1.22) 1.00 (0.91, 1.11) 0.96 (0.85, 1.07)	1.00 1.04 (0.84, 1.30) 1.43 (1.30, 1.57) 1.11 (1.00, 1.23) 1.45 (1.24, 1.70)	1.00 0.88 (0.69, 1.12) 1.51 (1.32, 1.73) 1.02 (0.88, 1.18) 1.56 (1.25, 1.95)
Treatment	Valproate Lamotrigine Topiramate	1.00 1.26 (1.18, 1.34) 1.61 (1.50, 1.73)	1.00 1.24 (1.14, 1.36) 1.29 (1.18, 1.42)	1.00 1.57 (1.38, 1.79) 2.16 (1.88, 2.47)

N/A – variable not included in multivariable model; Epi. abnorm. – epileptiform abnormality;

N/S abnorm. – non-specific abnormality; gen. TC – generalised tonic-clonic; myo./abs + TC – myoclonic or absence with tonic-clonic; unclass. TC – unclassified tonic-clonic

## Discussion

The PWP-TT model for focal epilepsy suggests that participants with a relative with epilepsy have a lower rate of recurrent seizures than people without such a relative, and that people with an abnormal CT/MRI scan results have a higher rate of recurrent seizures than those with a normal scan result. People with frontal lobe, other, or unclassified site of onset have a lower rate of recurrent seizures than people with temporal lobe site of onset. Additionally, people with simple or complex seizures with generalised tonic-clonic seizures, and people with uncertain seizure type have a higher rate of recurrent seizures than people with simple or complex partial seizures. Also, people with a higher rate of seizures before randomisation have a lower rate of recurrent seizures than those with a lower rate of seizures before randomisation. This final result is contrary to expectation but is due to an interaction with febrile seizure history (*p*-value: 0.03): the few people who had febrile seizures had higher pre-randomisation rates. This interaction term is not included in the model, as it vastly increases the complexity of the model interpretation.

The PWP-TT model for generalised epilepsy suggests that participants restarting treatment following seizures after remission have a lower rate of recurrent seizures than treatment naïve participants, that young participants (less than or equal to seven) have a lower rate of recurrent seizures than those aged eight or above, and that people with neurological insult have a higher rate of recurrent seizures than those without such an insult. Additionally, participants with febrile seizure history have a lower rate of recurrent seizure those who did not have such a history. Clinical intuition would suggest that participants with a febrile history seizure have a poorer clinical outcome. However, a history of febrile seizures is more often associated with focal epilepsy rather than generalised and unclassified as considered here [11, 22]. This, combined with the fact that only 8% of participants under consideration here had a history of febrile seizures, potentially explains this spurious finding.

The results of the Cox models have been discussed previously [11, 22]. While it is not appropriate to directly compare the results from the PWP-CTT model with those for conventional Cox models, we found that the results were fairly similar. Similarly the results of the negative binomial models. In general the direction of effect was consistent even if the significance of the covariate was not. Observed differences are likely to result from the number of variables included in the model, the underlying baseline hazard function, and the statistical power of the models. In particular, traditional Cox models consider a specific event with a fixed underlying intensity function while the PWP-TT model enables the underlying intensity function to vary from event to event [23].

The PWP-TT model accounts for all events along a patients’ journey and models time between each event. It also has improved statistical power over the Cox and negative binomial models. The PWP-TT model additionally estimates risk of future recurrent events rather than just time to a specified event. However, the data set-up is quite complex and the size of the dataset can be very large, especially for clinical conditions with many recurrent events such as seizures in epilepsy. Additionally the addition precision of the PWP-TT model is mirrored by a slight reduction in the ease of interpretation of the output.

A limitation of this analysis is the way the seizure data were collected within SANAD. Specifically, people were asked to report number of seizures since their previous appointment together with the date of the most recent seizure and first seizure since the last appointment. Therefore, dates of specific seizures were not collected. There is some evidence to suggest that seizures beget seizures [7]. However, we have not been able to investigate this further, specifically regarding treatment effect between the PWP-TT and negative binomial models, due to the limitations of this, and most routinely collected data within epilepsy trials.

Few published analyses of clinical data have utilised the PWP-TT model. Those that have include an analysis of diarrhoeal episodes in children [24], a population-based study of repetitive traumatic brain injury among persons with traumatic brain injury [25], recurrent malaria episodes [26], and childhood infectious diseases [6]. Such a model should be considered when designing future clinical trials in medical conditions typified by recurrent events, to ensure improve efficiency and statistical power as well as providing evidence regarding changes in event rates over time.

## Conclusions

Cox’s proportional hazard model is frequently used within the clinical literature to model time to a specified event. As demonstrated in this manuscript, this is useful for determining the initial response to treatment and potentially informing when the next intervention may be required. A variant on the Cox model, the PWP-TT, extends the Cox model to consider all events, not just the first. An alternative is the negative binomial model which considers event counts. We have shown the PWP-TT model to be useful to determine the longer-term effects of treatment policy. The PWP-TT model is therefore useful to increase understanding of chronic diseases.

Further work is now required to validate these epilepsy models in alternative data. The most relevant independent data will be the results from the SANAD II study which are not due to be released until the end of 2019 at the earliest.

## Data Availability

The datasets analysed during the current study are not publicly available as they contain information that could comprise the privacy of participants but are available from Professor Marson (A.G.Marson@liverpool.ac.uk) on reasonable request.

## Abbreviations

AEDs: Antiepileptic drugs
CT: Computerised tomography
EEG: Electroencephalogram
MRI: Magnetic resonance imaging
PWP-TT: Prentice, Williams and Peterson – Total Time
SANAD: Standard versus New Antiepileptic Drug
